# Prevention of Allogeneic Cardiac Graft Rejection by Transfer of *Ex Vivo* Expanded Antigen-Specific Regulatory T-Cells

**DOI:** 10.1371/journal.pone.0087722

**Published:** 2014-02-03

**Authors:** Fumika Takasato, Rimpei Morita, Takashi Schichita, Takashi Sekiya, Yasuhide Morikawa, Tatsuo Kuroda, Masanori Niimi, Akihiko Yoshimura

**Affiliations:** 1 Department of Microbiology and Immunology, Keio University School of Medicine, Tokyo, Japan; 2 Department of Pediatric Surgery, Keio University School of Medicine, Tokyo, Japan; 3 Department of Pediatric Surgery, International University Medical Welfare Hospital, Nasushiobara, Tochigi, Japan; 4 Department of Surgery, Teikyo University School of Medicine, Tokyo, Japan; 5 Japan Science and Technology Agency, CREST, Tokyo, Japan; University of Tokyo, Japan

## Abstract

The rate of graft survival has dramatically increased using calcineurin inhibitors, however chronic graft rejection and risk of infection are difficult to manage. Induction of allograft-specific regulatory T-cells (Tregs) is considered an ideal way to achieve long-term tolerance for allografts. However, efficient *in vitro* methods for developing allograft-specific Tregs which is applicable to MHC full-mismatched cardiac transplant models have not been established. We compared antigen-nonspecific polyclonal-induced Tregs (iTregs) as well as antigen-specific iTregs and thymus-derived Tregs (nTregs) that were expanded via direct and indirect pathways. We found that iTregs induced via the indirect pathway had the greatest ability to prolong graft survival and suppress angiitis. Antigen-specific iTregs generated *ex vivo* via both direct and indirect pathways using dendritic cells from F1 mice also induced long-term engraftment without using MHC peptides. In antigen-specific Treg transferred models, activation of dendritic cells and allograft-specific CTL generation were suppressed. The present study demonstrated the potential of *ex vivo* antigen-specific Treg expansion for clinical cell-based therapeutic approaches to induce lifelong immunological tolerance for allogeneic cardiac transplants.

## Introduction

Transplantation is the ultimate treatment for patients with total loss of function of a life-sustaining organ. New immunosuppressive drugs have improved allograft survival rates, but long-term administration of these agents may have serious side-effects, including nephrotoxicity, diabetes, neurotoxicity, and increased risk of infection and cancer [1]–[3]. These complications could be avoided by establishing a technique for donor-specific unresponsiveness or immunologic tolerance to donor alloantigens in transplant recipients. Active suppression by regulatory T cells (Tregs) is likely ideal for the induction of tolerance to allografts. Several attempts have been made; however, methods for the induction of lifelong tolerance to specific alloantigens have not been established.

We previously reported that systemic injection of an allopeptide, a 15-mer (54–68), corresponding to a hypervariable region of the K^b^ molecule to CBA (H2^k^) mice prolonged the survival of a cardiac graft of a C57BL/10 (H2^b^) but not third party BALB/c (H2^d^) hearts [4], [5]. Adoptive transfer of splenocytes from mice pretreated intratracheally with the K^b^ peptide to naïve secondary recipients also prolonged the survival of cardiac grafts, suggesting the generation of allograft antigen-specific regulatory cells. Thus, generation of Tregs specific to the K^b^ peptide e*x vivo* as well as adoptive transfer could achieve successful prevention of cardiac allograft rejection.

Dendritic cells (DCs) are the most potent antigen-presenting cells (APCs), with the unique ability to activate or suppress adaptive immune responses depending on maturation status and cytokine production. “Tolerogenic DCs” or immunoregulatory DCs have been characterized as Treg-inducing cells. These tolerogenic DCs are usually immature, expressing reduced levels of MHC molecules, co-simulators, and inflammatory cytokines but enhanced levels anti-inflammatory cytokines such as IL-10 and TGF-β. It is well established that tolerogenic DCs induce Foxp3^+^ Tregs through a TGF-β dependent mechanism. Foxp3^+^ Tregs can be generated from naïve T cells by T cell receptor (TCR) stimulation in the presence of TGF-β and IL-2, so called induced Tregs (iTregs). iTregs have similar suppression activity *in vitro* to thymus-derived naturally occurring Tregs (nTregs). However, Foxp3 expression of iTregs was believed to be unstable *in vivo* under lymphopenic conditions [6]–[8]. Several methods have been proposed to generate antigen-specific Tregs *in vitro.* All-trance retinoic acids (ATRAs) have been shown to enhance and stabilize Foxp3 expression [9], [10]. Experimental generation of tolerogenic DCs has been accomplished through treatment with maturation-inhibiting agents, blockade of costimulatory molecules, either with antibodies or antisense oligonucleotides, as well as pretreatment with chemical immunosuppressants [11]–[15].

Cellular therapy with CD4^+^CD25^+^ Tregs is limited because of the inability to consistently generate and expand antigen-specific suppressors *in vitro*. Several strategies have been attempted for *ex vivo* propagation of Tregs; however, *in vitro* generated Tregs by tolerogenic DCs could only partially prevented rejection of an allogenic heart graft [16]. Joffre et al have generated *ex vivo*-expanded allograft-specific Tregs by co-culturing with DCs from F1 mice[17]. This method can induce Tregs via both direct and indirect pathways because F1 DCs are expected to express allogenic MHC molecules as well as self-MHCs carrying antigenic MHC peptides. However, this group utilized *in vivo* bone marrow transplantation into irradiated mice to generate Tregs. Therefore, *in vitro* method for the generation of alloantigen-specific Treg is desired to be established. At least two distinct pathways of allorecognition exist: direct and indirect pathways. In the direct pathway, host T cells recognize intact allo-MHC molecules on the donor APCs. In the indirect pathway, T cells recognize processed alloantigen presented as peptides by self-APCs.

In this study, we have tried to establish methods to generate alloantigen specific Tregs to induce long-term tolerance to MHC full-mismatched cardiac grafts. We found that iTregs expanded by peptide-loaded isogenic DCs (indirect pathway) could induce long-term tolerance to MHC full-mismatched cardiac grafts. In contrast, iTregs expanded by allogenic DCs (direct pathway) could prevent acute rejection but not chronic rejection. We also compared nTregs and iTregs expanded by the indirect pathway and found that iTregs were more effective than nTregs in suppressing chronic inflammation. We observed an immunosuppressive feedback loop by DC, Tregs, and CTLA4^+^CD8^+^ T cells in the graft. We established a simple and effective protocol to generate alloantigen-specific Tregs that can induce long-term immunological tolerance.

## Materials and Methods

### Mice

Male C57BL/6 (H-2K^b^), CBA/N (H-2K^k^), and BALB/c (H-2K^d^) mice (6–10 weeks old) were purchased from Sankyo Ltd. (Tokyo, Japan), housed in conventional facilities at the Biomedical Services Unit of Keio University (Tokyo, Japan). Mice were kept in conventional conditions in Keio University (Tokyo, Japan). All experiments using these mice were approved by Institutional Animal Care and Use Committee (IACUC) (approved number 08004) of Keio University and performed according to the guidelines of IACUC. All experiments using these mice were approved by and performed according to the guidelines of the Animal Ethics Committee of Keio University. The donor mouse was anaesthetized with an intraperitoneal injection of pentobarbital sodium 45 mg/kg. The donor mouse was euthanized by cervical dislocation under anesthesia.

### Kb Peptide

K^b^ peptide 54–68 (QEGPEYWERETQKAKG) of class I MHC H2-K^b^ was used to induce antigen-specific Tregs [4]. The peptide was prepared by chemical synthesis (BEX Corporation Ltd., Tokyo, Japan; SCRUM Corporation Ltd., Tokyo, Japan).

### Antibodies

FITC-, PerCP-Cy5.5-, APC-, and PE-conjugated monoclonal antibodies for CD3 (17A2), CD4 (RM4-5), CD25 (PC61.5), CD11c (N418), CD8 (53-6.7), Foxp3 (FJK-16s), CTLA4 (UC10-4B9), CD80 (16-10A1), CD86 (GL-1), CD40 (1C10), CD40L (MR1), CD103 (2E7), I-A/I-E (M5/114.15.2), IFN-γ (XMG1.2), IL-6 (MP5-20F3), Granzyme B (GB11-Biolegend), Perforin (eBioOMK-D), and IL-10 (JES5-16E3) antibodies were purchased from eBioscience. Before IFN-γ, IL-6, and IL-10 intracellular staining, cells were stimulated with 50 nM PMA (Sigma-Aldrich, St. Louis, MO, USA), 1 µg/ml ionomycin (Sigma-Aldrich), and 1 µM Brefeldin A (eBioscience) for 4.5 h. After surface staining for 30 min, the cells were suspended in Fixation Buffer (eBioscience), and intracellular cytokine staining was performed as described [18].

### Heart Transplantation

Fully vascularized heterotopic hearts from C57BL/6 donors were transplanted into CBA/N recipients using microsurgical techniques as previously described [19]. In some experiments, CBA mice were used as recipients, and BALB/c or C57BL/6 mice were used as donors of cardiac allografts. Postoperatively, the graft function was assessed daily by palpation for evidence of contraction. Rejection was defined as complete cessation of contraction and confirmed by direct visualization of the graft.

### Histologic Studies of Harvested Grafts

Cardiac allografts in untreated mice and mice given Tregs were removed 7, 14–15, 35–40, and 120 days after transplantation and studied histologically. The specimens were fixed with 4% paraformaldehyde phosphate buffer solution and embedded in paraffin using routine procedures. Paraffin sections (4 µm thick) were cut, mounted on saline-coated slides, and stained with hematoxylin-eosin (HE).

### Preparation of Naïve CD4^+^T Cells, nTregs, and Naïve CD8^+^ T Cells

Spleen and lymph nodes were harvested from CBA mice. Cells were harvested from spleen and lymph nodes and separated using a MACS separator (Miltenyi Biotec, Tokyo, Japan) with both negative selection and positive selection. The purified cells were then used for the experiments, except for nTregs that were separated additionally using fluorescence-activated cell sorting FACSAria (BD Biosciences, Tokyo, Japan) and CD3^+^CD4^+^CD25^+^ cells were collected and used in the experiments as described [20], [21]. The purity of the Tregs was determined by the percentage of CD3^+^CD4^+^CD25^+^Foxp3^+^ cells using anti-Foxp3 antibody staining [22]. For naïve CD8^+^ T cells, the purified CD8^+^ T cells were further separated with anti-CD62L MicroBeads (Miltenyi Biotec) and the MACS column. The purity of the naïve CD8^+^ T cells was >95%.

### Preparation of Bone Marrow-Derived Dendritic Cells (BMDCs)

BMDCs were prepared as described previously [23], [24]. Briefly, BM cells harvested from the femurs and tibia were cultured in 10 cm dishes (2x10^6^/10 ml/dish) in complete medium containing RPMI 1640 supplemented with 10% fetal bovine serum (FBS; Invitrogen, Carlsbad, CA, USA), 1% penicillin/streptomycin, 0.05 mM 2-mercaptoethanol (ME) (Invitrogen), and 10 ng/ml murine GM-CSF (Pepro Tech). At day 4, another 10 ml of complete medium containing 10 ng/ml GM-CSF was added to the plates. At day 6 and day 8, half of the culture supernatant was collected, and the dose of complete medium with 10 ng/ml GM-CSF was added. Days 8–10, non-adherent cells were collected and used for the experiment. The purity of the CD11c^+^ cells was >90% based on flow cytometry. For mature DCs, BMDCs were reseeded in 12-well plates (1×10^6^/well) on day 8 with complete medium and were stimulated with 10 ng/ml lipopolysaccharide (LPS) (*Escherichia coli* serotype 055:B5, Sigma-Aldrich) for 20–22 h.

### Induction of Antigen-Specific iTregs and nTregs

For Tregs expanded via the indirect pathway, purified naïve T cells (5×10^4^/well) from CBA mice were cocultured with BMDCs (5×10^4^/well). For iTregs, cells were incubated in complete medium with 5 µg/ml anti- IFN-γ Ab (R4-6A2), 5 µg/ml anti-IL-4 Ab, 2 ng/ml human TGF-β (R&D systems), 10 ng/ml IL-2 (PeproTech), 20 nM all-trans retinoic acid (ATRA; Sigma), and 10 µg/ml K^b^ peptides using 96-well U-bottom plates. On day 6, half of the medium was changed. Cells harvested on days 8–10 were used for the experiment. For nTregs, CD3^+^CD4^+^CD25^high^ cells were incubated in the same medium as the iTregs, except 100 ng/ml IL-2. On day 6, we reseeded them in 4-fold complete medium, with the same cytokines and BMDCs.

For iTreg induction via the direct pathway, purified naïve T cells (5×10^4^/well) from CBA mice were cocultured with BMDCs (2.5×10^4^/well) from C57BL/6 mice. The cells were incubated in the same medium as the indirect pathway iTregs, except with the addition of 10 µg/ml anti- IFN-γ Ab. On days 5–6, the cells were used for the experiment. For antigen-nonspecific iTreg Induction, naïve CD4^+^ T-cells were stimulated with anti-mouse CD3 Ab and anti-CD28 Ab in the presence of 10 ng/ml IL-2, 20 nM ATRA, and 2 ng/ml human TGF-β for 3 days as described [25].

Before injection, the cultured cells were labeled with anti-CD3, anti-CD4, and anti-CD25 antibodies, and the CD3^+^CD4^+^CD25^high^ cells were then sorted using FACSAria. The Treg purity was >95% judged by anti-Foxp3 antibody staining. Then, 1×10^6^ Tregs/mouse were injected intravenously.

### Isolation of Cardiac Graft Leucocytes

After perfusion using normal saline, cardiac grafts were excised and washed in ice-cold normal saline. The grafts were minced into 2–3-mm pieces and digested with 1 mg/ml collagenase D (Roche) and 0.25 mg/ml DNaseI in RPMI 1640 with 10% FBS for 30 min at 37°C with constant agitation. The remaining tissues were further minced, and lymphocytes were purified on a 37%/75% Percoll (GE Healthcare) gradient by centrifugation at 2000 rpm for 25 min at 25°C. Infiltrated mononuclear cells were analyzed by FACS as described [26], [27].

### Statistical analyses

All data are expressed as the mean ± standard error (SE). The data were statistically analyzed using the Log-rank test, Tukey's test, Bonferroni test, and the independent-sample *t*-test. *P*<0.05 was considered statistically significant.

## Results

### Optimization of Induction of Antigen-Specific Regulatory T Cells

We have tried to establish methods to expand donor-antigen-specific Tregs *in vitro*. We transplanted hearts from donor C57BL/6 mice (H-2K^b^) into host CBA (H-2K^k^) mice. To induce allo-specific T cells via the direct pathway, T cells from CBA mice were stimulated with BMDCs from C57BL/6 mice without any antigens. To induce alloantigen-specific T cells via the indirect pathway, T cells from CBA mice were cultured with BMDCs from CBA mice loaded with the 15-mer (54–68) H-2K^b^ (K^b^) peptide which is located in a hypervariable region of the K^b^ molecule of the MHC class I of C57BL/6 mice. This peptide was chosen because we have shown that intratracheal delivery of this peptide induced hyporesponsiveness to allogeneic cardiac grafts and generated regulatory cells [4].

It has been shown that Foxp3^+^ iTregs can be induced from naïve T cells by co-culture with immature DC in the presence of IL-2 and TGF-β [28]–[31]. To establish the best conditions for indirect pathway-mediated expansion of alloantigen-specific iTregs, the BMDC/naïve T-cell ratio, peptide concentration, and cytokine combination were examined.

**Figure 1:**
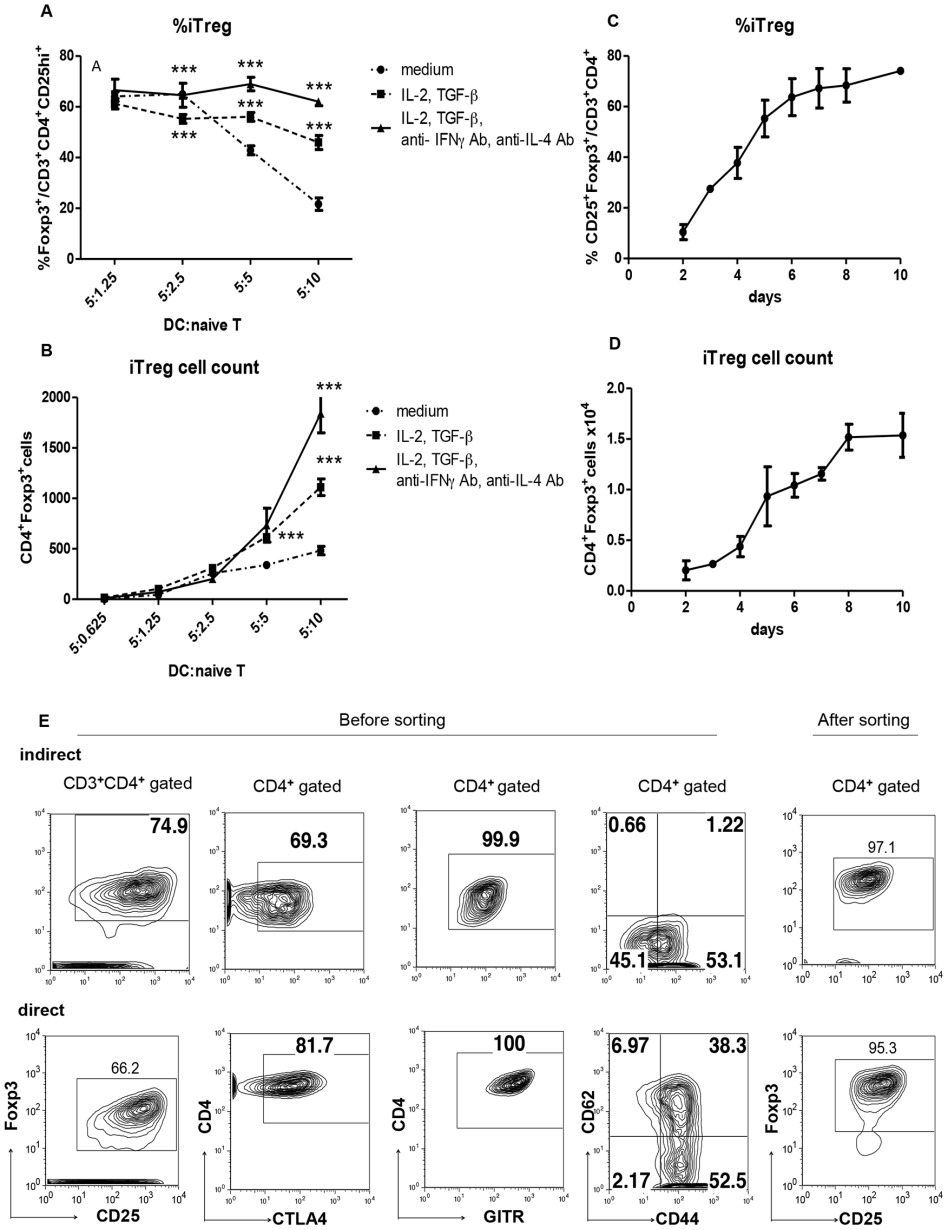
Expansion of Tregs by BMDCs *in vitro*. **A, B,** *In vitro* antigen-specific expansion of iTregs via the indirect pathway. Naïve CD4^+^ T cells from CBA mice were cultured with BMDCs (CBA) in the presence of the K^b^ peptide for 7 days at the indicated conditions. The Treg fraction was determined as CD3^+^CD4^+^ CD25^+^Foxp3^+^ (***P<0.001, Bonferroni test) **C, D,** The-time course of Treg fraction (C) and number (D) in the *in vitro* culture with BMDCs in the presence of 10 µg/ml K^b^ peptide, 10 ng/ml IL-2, 2 ng/ml TGF-β, 5 µg/ml anti- IFN-γ and anti-IL-4 antibodies, and 20 nM ATRA. **E,** The cell surface marker and purity of the Tregs before and after cell sorting, on day 8 after culture via the indirect and direct pathway. Tregs induced via the direct pathway were cultured with C57BL/6 BMDCs and 10 ng/ml IL-2, 2 ng/ml TGF-β, 10 µg/ml anti- IFN-γ and 5 µg/ml anti-IL-4 antibodies, and 20 nM ATRA. The cells were stained with the indicated antibodies and analyzed with FACS.

The Treg expansion was assessed with CD25- and Foxp3-positivity using fluorescence-activated cell sorting (FACS). First, naïve CD4^+^ T cells were cultured with various numbers of BMDCs in the presence of a fixed amount of peptide, IL-2, and TGF-β. The effect of anti- IFN-γ Ab and anti-IL-4 Ab was also examined (**Fig. 1A, B**). Although the number of Foxp3^+^ Tregs increased as the BMDC number increased, the purity of the Tregs (Foxp3^+^CD25^high^/CD4^+^) decreased. Therefore, we chose a 1∶1 ratio (each 5×10^4^ cells/well) for optimal iTreg induction. The induction was improved when including anti- IFN-γ and anti-IL-4 antibodies (**Fig. 1A, B**). For the starting cell number, a culture of more than 8×10^4^ T cells per well resulted in an increase number of dead cells and a decreased Treg purity. Therefore, we chose a starting cell number of 5×10^4^ T cells/well per culture. We also found that 8 days was the best culture period to obtain the highest number of iTregs (**Fig. 1C, D**). Radiation of BMDCs did not affect iTreg induction, while activation of BMDCs with LPS strongly reduced iTreg induction (data not shown). Thus, we used fresh BMDCs for the T cell expansion. We also examined various peptide concentrations and concluded that 10 µg/ml peptide was the most efficient for antigen-specific Treg induction. We also found that 20 nM ATRA increased the Treg purity to 95%. In addition, half of the medium was changed to fresh medium on day 6, and the cells were further cultured to days 8–10 (**Fig. 1C, D**). After co-culture with BMDCs, the CD3^+^CD4^+^CD25^high^ fraction was sorted with FACS (**Fig. 1E**). Such optimized conditions (DC:T  = 1∶1, 10 ng/ml IL-2, 2 ng/ml TGF-β, 5 µg/ml anti- IFN-γ and 5 µg/ml anti-IL-4 antibodies, and 20 nM ATRA) enabled us to obtain more than 1×10^6^ iTregs with >95% purity, which is necessary for the transfer to one mouse (**Fig. 1E**). These cells were designated indirect-iTregs.

To induce allogenic iTregs by the direct pathway, BMDCs from C57BL/6 mice and naïve T cells from CBA mice were co-cultured. The best condition was 1∶2 DC/naïve T-cell ratio, culture in the presence of IL-2, TGF-β, and ATRA, together with anti- IFN-γ and anti-IL-4 antibodies. Then, the CD3^+^CD4^+^CD25^high^ fraction was sorted with FACS (Foxp3 positivity >95%), and these cells were designated direct-iTregs (**Fig. 1E**). CTLA4^+^ and GITR^+^ expression in direct-iTregs was slightly higher than that of indirect-iTregs, although two types of Tregs showed similar CD62L^low^ activated phenotypes.

### iTregs Induced by the Indirect Pathway but Not by the Direct Pathway Efficiently Prevented Chronic Cardiac Graft Rejection

**Figure 2:**
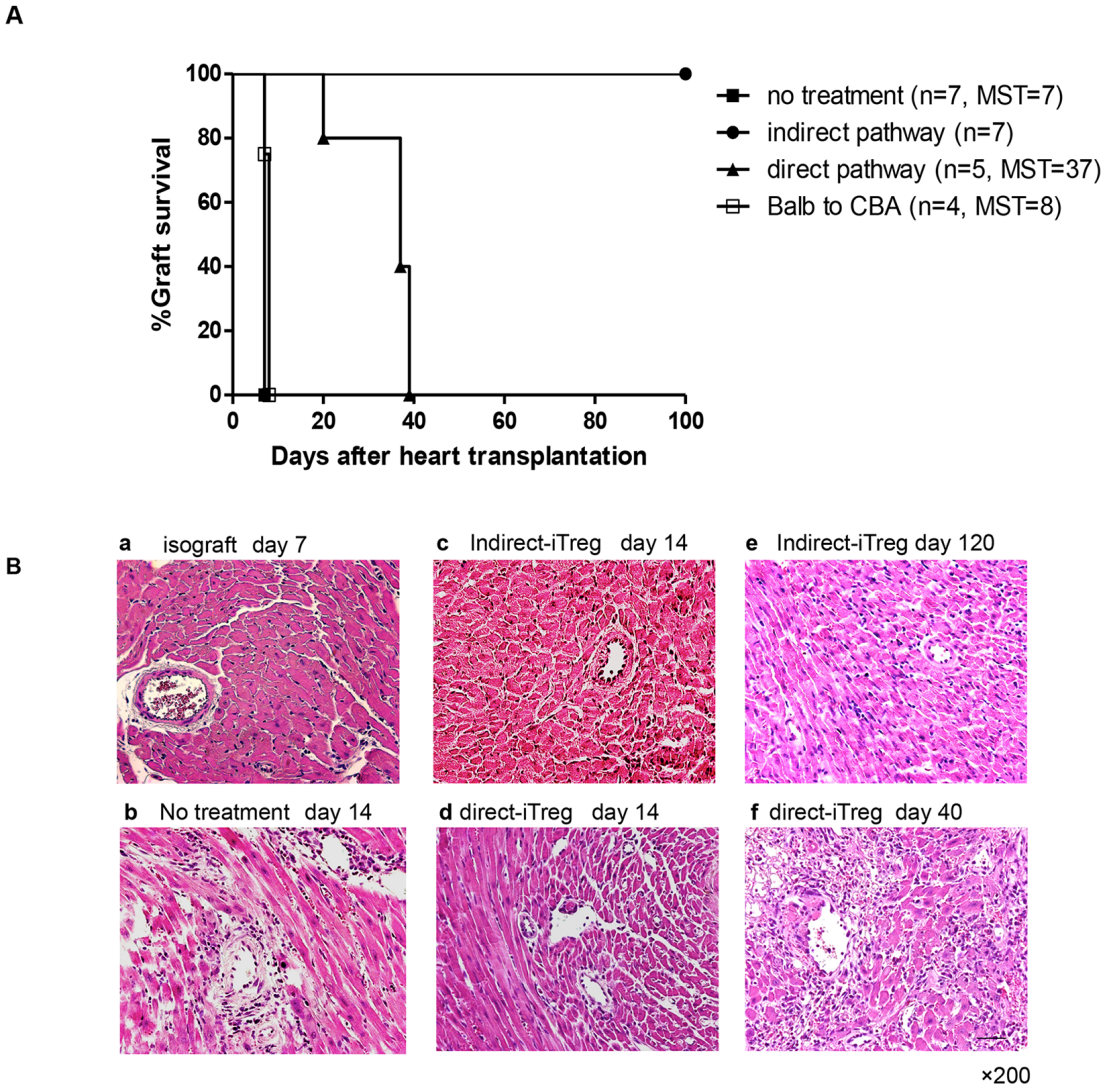
Prevention of graft rejection by Tregs. **A,** Cardiac graft survival in mice treated with antigen-specific iTregs (CBA background) induced by the indirect pathway and direct pathway. Tregs (1×10^6^ cells) were injected intravenously one day before transplantation of the C57BL/6 heart into CBA recipients. BALB/c to CBA cardiac transplantation was performed after injection of indirect-iTregs (CBA) against the K^b^ peptide. (The Log-rank test was used; *P* values are compared between the indirect pathway and other pathways; no treatment, *P*<0.001; anti-CD3 Ab/anti-CD28 Ab, *P*<0.001; BALB/c vs. CBA, *P* = 0.0013; direct pathway, *P*<0.001. Direct-iTreg treatment vs. no treatment, *P*<0.001) **B,** HE staining of harvest cardiac grafts indicated days after transplantation. The bar of the right lower panels was 10 µm. Magnification; 200×.

First, we examined which type of iTreg, direct-iTreg, or indirect-iTreg effectively prevented cardiac graft rejection. As shown in **Fig. 2A**, naïve CBA mice rejected C57BL/6 cardiac grafts acutely (median survival time [MST]  = 7 days). Indirect-iTregs extensively prolonged the graft survival, >100 days, while direct-iTregs showed a partial effect (MST  = 37 days). Thus, iTregs expanded by a specific MHC class I peptide presented on self-DCs were more effective than those induced by heterogeneous alloantigens on allogeneic DCs. Moreover, third-party BALB/c cardiac grafts were rejected in recipients pretreated with indirect-iTregs induced by the K^b^ peptide (MST  = 8 days) (**Fig. 2A**), indicating that the tolerance induced by indirect-iTregs was antigen specific and that the possibility of non-specific immunosuppression by a bystander effect of Tregs could be eliminated.

Histological examination showed all typical signs of chronic cardiac allograft rejection, including diffuse cell filtration and destruction of cardiac myocytes, which were observed in non-treated grafts on day 14 after transplantation (**Fig. 2B** **b**). In contrast, little or no obvious signs of cardiac myocyte destruction were observed in grafts administered indirect-iTregs on day 14 (**Fig. 2B** **c**). Direct-iTreg treatment also suppressed cardiac myocyte destruction; however, infiltration of mononuclear cells was apparently observed on day 14 (**Fig. 2B** **d**). No obvious myocyte destruction, except for a mild angiitis, was observed in grafts in mice treated with indirect-iTregs, even 120 days after transplantation (**Fig. 2B** **e**). However, the grafts in mice treated with direct-iTregs showed partial fibrosis, destruction of myocytes, and bleeding on day 40 after transplantation, confirming that direct-iTregs could not suppress chronic rejection. Thus, direct-iTregs had much weaker potential of tolerance induction than indirect-iTregs (**Fig. 2B** **f**).

### Comparison of iTregs and nTregs Expanded by the Indirect Pathway

**Figure 3:**
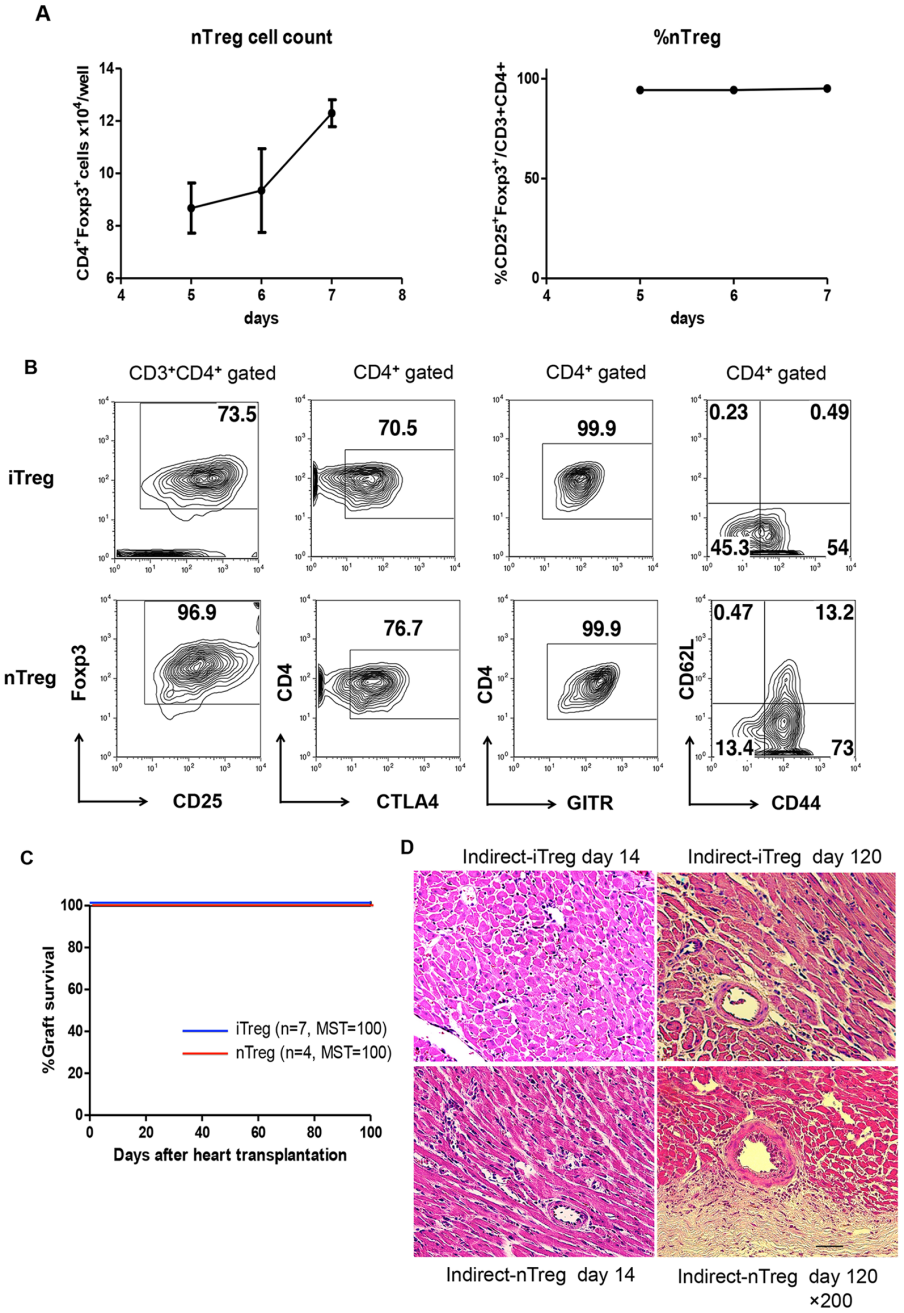
The effect of nTregs expanded by the indirect pathways. **A,** Expansion of alloantigen-specific nTregs *in vitro*. nTregs (5×10^4^) from CBA were cultured with 5×10^4^ allo-BMDCs in the presence of 10 µg/ml K^b^ peptide, 100 ng/ml IL-2, 2 ng/ml TGF-β, 5 µg/ml anti- IFN-γ and anti-IL-4 antibodies, and 20 nM ATRA, and the number (left) and fraction (right) of Tregs were estimated. **B,** Comparison of cell surface markers of iTregs and nTregs expanded by the indirect pathway for 8 days before cell sorting. **C,** Effect of iTregs and nTregs on graft survival in cardiac transplantation models. **D,** HE staining of harvested cardiac grafts.

Next, we compared the effects of iTregs and nTregs on tolerance induction for cardiac allografts. CD4^+^CD25^high^ T cells were isolated from CBA mice and co-cultured with auto-BMDCs in the presence the K^b^ peptide, with the same procedure for iTregs. Because proliferation of nTregs required a higher concentration of IL-2, 100 ng/ml IL-2, instead of 10 ng/ml, was used for nTreg stimulation. Half of the medium was changed on day 6, and the cells were harvested on days 10–12. The CD3^+^CD4^+^CD25^high^ fraction of indirect-nTregs was >95% before sorting (**Fig. 3B**); thus, the purity of Tregs in the indirect-nTreg fraction was higher than that of indirect-iTregs after culture with BMDCs. The expression levels of CTLA4 and GITR were similar between iTregs and nTregs, although the CD44^high^CD62L^low^ fraction of indirect-nTregs was higher (**Fig. 3B**). After sorting, the Foxp3^+^ fraction was >95% in both the indirect-iTregs and indirect-nTregs (data not shown).

The nTregs and iTregs were then adoptively transferred to CBA mice, and cardiac transplantation was performed 24 h after transfer. As shown in **Fig. 3C**, both the nTregs and iTregs induced strong tolerance to allogenic cardiac grafts, and all grafts survived more than 100 days. However, a small area of destroyed cardiac myocytes associated with infiltration of mononuclear cells around the small blood vessels was frequently found in grafts of mice administered with indirect-nTregs on day 14 (**Fig. 3D**). While more severe vascular hypertrophy was observed in the grafts administered indirect-nTregs than in those with indirect-iTregs on day 120 after transplantation (**Fig. 3D**). These data indicate that indirect-nTregs had weaker potential for the induction of tolerance than indirect-iTregs, although the grafts survived >100 days after transplantation, as judged by heart beating.

### Characterization of Tregs infiltrated into Cardiac Grafts

**Figure 4:**
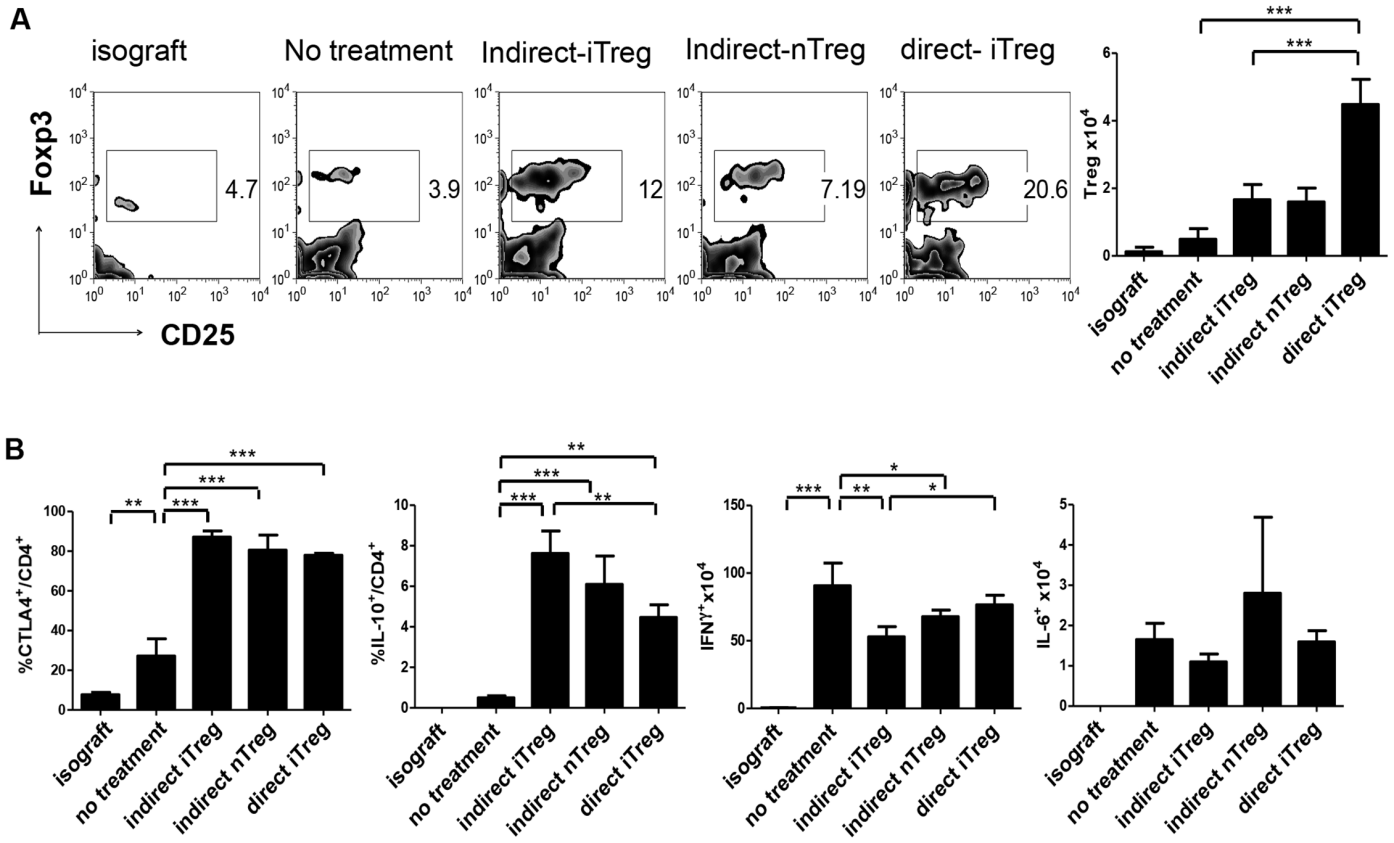
Tregs features in the cardiac grafts on day 7 after transplantation(n = 3–5). **A,** Proportion and cell number of Tregs in each graft. Mononuclear cells were separated from the graft by collagenase treatment and Percoll gradient centrifugation. After staining with CD3, CD4, CD25 and Foxp3 antibodies, cells were analyzed with FACS. In the lower panel, the number of infiltrated Tregs per graft is shown (n = 5). **B,** Mononuclear cells in the grafts were stained with anti-CTLA4 (left) and anti-IL-10 (left) antibodies after PMA, ionomycin, and brefeldin treatment, and analyzed with FACS (n = 3–5). The proportion of CTLA4- and IL-10-producing cells in the CD4^+^ T cell population in the grafts is shown. **C,** The cell number of IFN-γ and IL-6-producing cells. The mononuclear cells infiltrated into the grafts were stained with anti- IFN-γ and anti-IL-6 antibodies and analyzed with FACS (n = 3–5). (*** *P*<0.01, ** *P*<0.03, * *P*<0.05, Tukey's Multiple Comparison Test).

To investigate the mechanism of prevention of allograft rejection by Treg adoptive transfer, we compared the infiltrated Tregs cells on day 7 in the cardiac grafts with the Treg-treated recipient mice. Mononuclear cells were isolated from collagenase-treated grafts by Percoll gradient centrifugation and analyzed with FACS. As shown in **Fig. 4A**, the number of Foxp3^+^CD4^+^ Tregs in the graft on day 7 increased in Treg-treated mice compared with untreated mice. Notably, not only the number but also the fraction of Tregs drastically increased in mice treated with direct-iTregs compared with indirect-iTregs or nTregs. This is consistent with the previous notion that acute organ graft rejection has been attributed mainly to direct antigen presentation [32].

We compared the expression levels of effector molecules of Tregs (**Fig. 4B**). Most CD4^+^ T cells in the grafts of the mice treated with all types of Tregs expressed high levels of CTLA4. IL-10-positive cells were also increased in graft-associated CD4^+^ T cells in all Treg-treated mice, although the IL-10^+^ fraction was lower in direct-iTreg-treated mice than in indirect-Treg-treated mice. Associated with the increase in IL-10-producing Tregs, the number of IFN-γ^+^ T cells, which play important roles in chronic rejection, decreased by Treg administration (**Fig. 4C**). However, IL-6 expression did not correlate with the severity of the rejection (**Fig. 4C**).

### Induction of immature DCs and CTLA4^+^CD8^+^ Tregs in the Grafts with Treg Therapy

**Figure 5:**
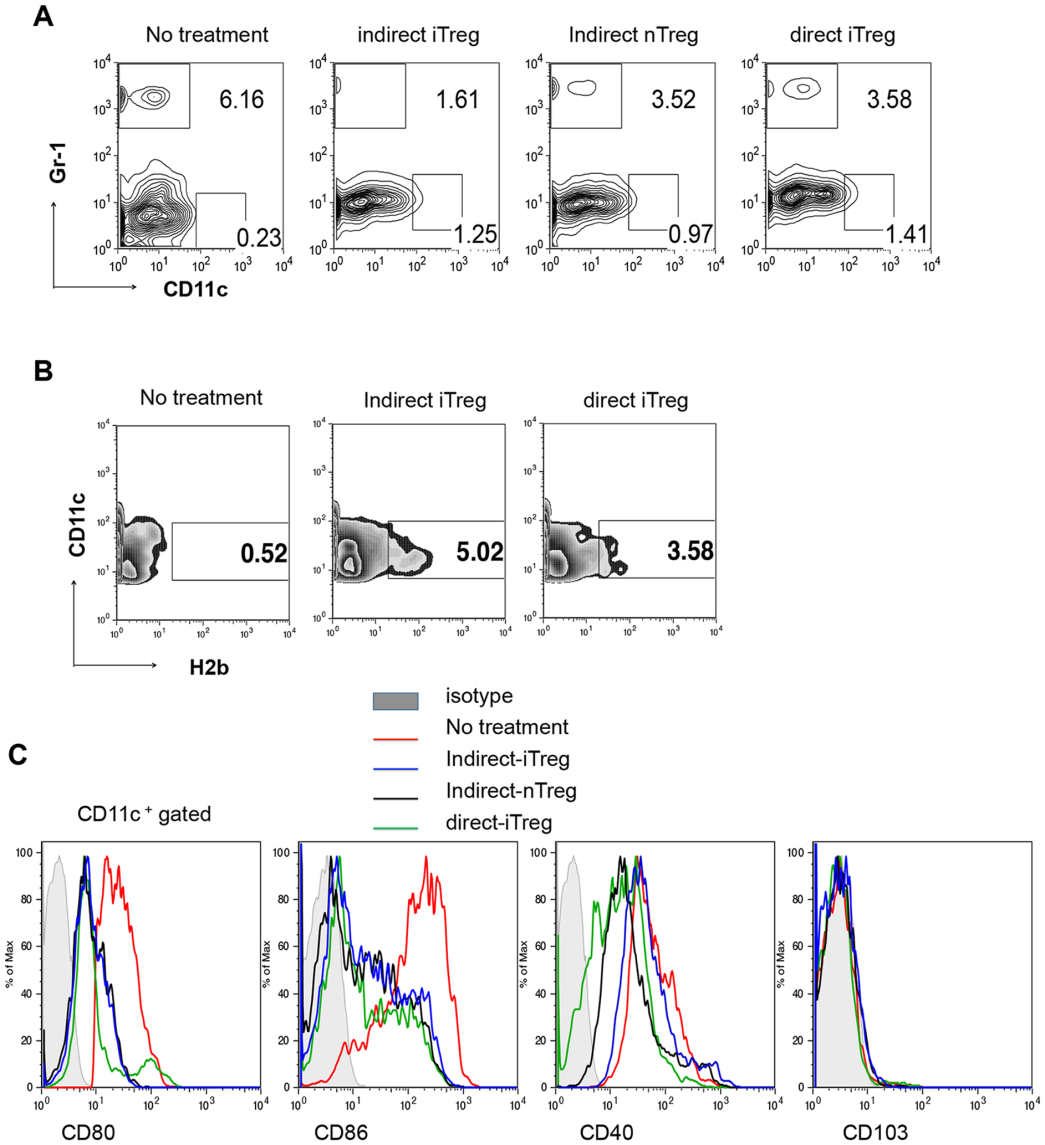
Analysis of DCs in the grafts. **A,** Gr-1 and CD11c staining in cells filtrated in cardiac grafts 7 days after transplantation in mice with the indicated treatment. **B,** Population of CD11c^+^H2b^+^ cells in the filtrated cardiac graft cells. The graft undergoing no treatment was harvested 14 days after transplantation, and other 2 grafts were harvested 35–40 days after transplantation. **C,** Cell-surface marker expression of CD11c^+^ cells filtrated in the cardiac grafts 14 or 15 days after transplantation in mice tread with the indicated Tregs.

As shown in **Fig. 5A**, infiltration of Gr1^+^ granulocytes was strongly reduced by indirect-iTreg treatment. Direct-iTreg and indirect-nTreg treatments also reduced granulocyte infiltration but were less effective than indirect-iTreg treatment. There is no strong correlation between F4/80-positive macrophage/monocyte infiltration among the Treg treatments (data not shown); however, CD11^+^ DCs were increased by the Treg treatment. Thus, we compared the nature of the DCs in the grafts of the mice treated with or without Tregs. Interestingly, as shown in **Fig. 5B**, donor-derived H2b^+^CD11c^+^ cells were present in the grafts in mice treated with iTregs, suggesting that Treg treatment increased the survival of graft DCs. While CD80 and CD86 co-stimulator and CD40 expression levels were lower in Treg-treated graft DCs than in non-treated graft DCs (**Fig. 5C**). There was no upregulation of CD103. These data suggest that transferred Tregs accumulated in the graft, thereby suppressing DC activation.

**Figure 6:**
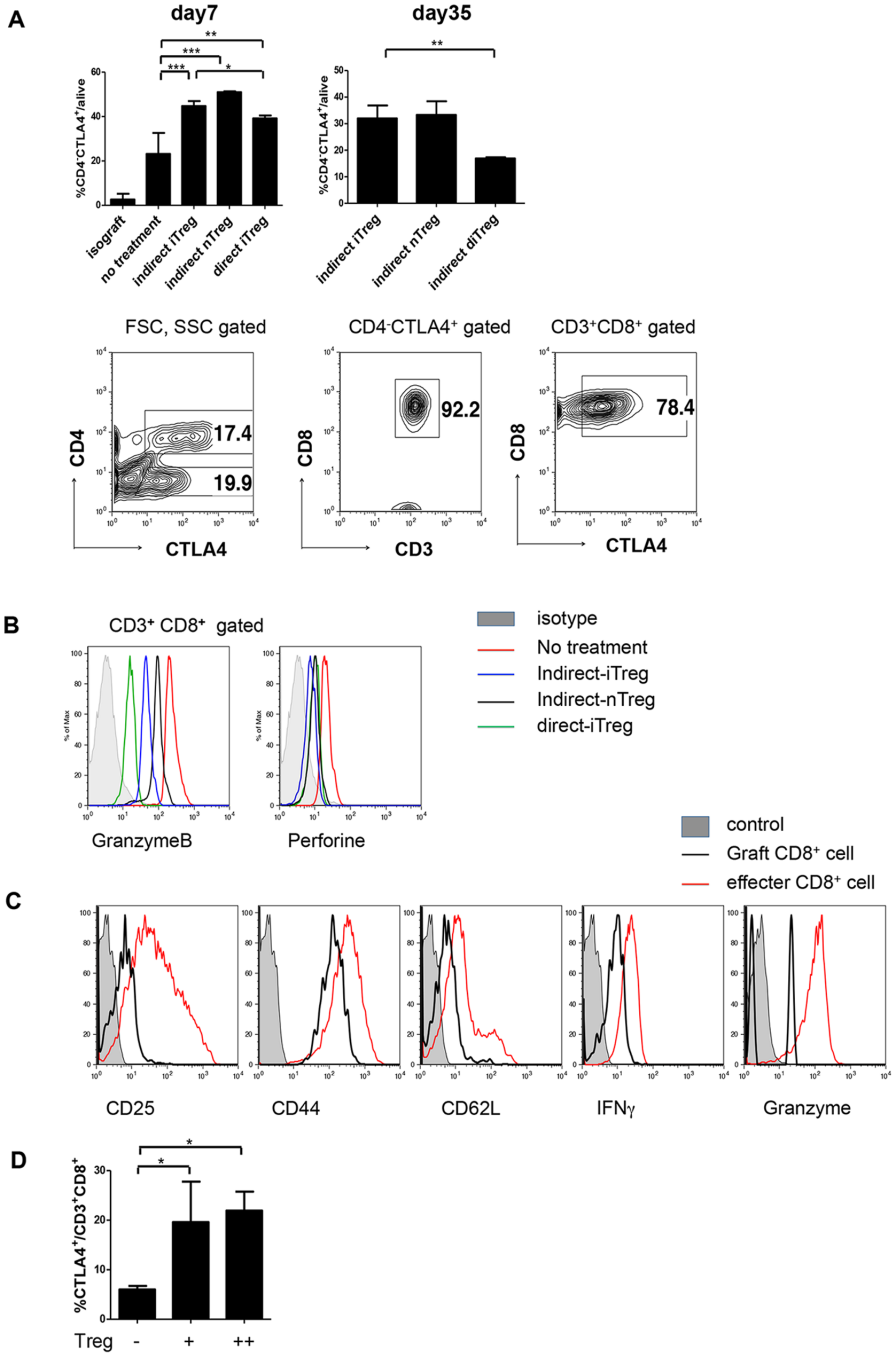
CTLA4^+^CD8^+^ cells in filtrated cardiac grafts. **A,** (upper panels) Fraction of CD4^−^CTLA4^+^ cells in mononuclear CD3^+^ cells in the grafts on day 7 and days 35–40 after transplantation (n = 3–5). *** *P*<0.01 ** *P*<0.03 (lower panels) FACS analysis of CTLA4^+^ cells in the graft of indirect-iTreg-treated mice on day 35 after transplantation. **B,** Histograms of CD3^+^CD8^+^ cells harvested from cardiac grafts 14 or 15 days after transplantation in mice treated with the indicated Tregs. **C,** Comparison of the expression of cell surface marker and cytokines between infiltrated CD8^+^ cells in indirect-iTreg-treated grafts on day 35, and effector CD8^+^ cells induced *in vitro*. Effector CD8^+^ cells were induced from naïve CD8^+^ T cells from CBA cultured with BMDCs from C57BL/6 mice (MLR). **D.** Induction of CD8^+^CTLA4^+^ cells. Using 96-well U-bottom plates, naïve CD8^+^ T-cells (2×10^4^/well, CBA) were cultured with 15 Gy-irradiated BMDCs (2×10^4^/well, C57BL/6) in either the presence or absence of direct-iTregs induced *in vitro* (4×10^4^/well (+) or 8×10^4^/well (++), CBA), as described above. After 5 days, the cells were analyzed using a FACSCanto. (* *P*<0.05, Bonferroni's Multiple Comparison Test).

Not only CD4^+^CTLA4^+^ cells (**Fig. 4B**) but also CD4^−^CTLA4^+^ cells (**Fig. 6A** **left**) drastically increased via the 3 types of Treg treatments. The CD4^−^CTLA4^+^ cells were maintained in the day 35 grafts in the mice treated with indirect-iTregs and indirect-nTregs; however, the this fraction decreased in grafts of mice treated with direct-iTregs (**Fig. 6A** **right**), suggesting that the CTLA4 levels correlated with the prevention of chronic rejection. As show in **Fig. 6A** **lower panels**, these were CD8^+^ T-cells. CD8^+^ T-cells in the graft treated with Tregs showed reduced expression of Granzyme B and perforin molecules compared with those from untreated grafts (**Fig. 6B**). Moreover, in the grafts treated with indirect-iTregs, CD25^+^, IFNγ and Granzyme B in CD8^+^ T-cells were reduced compared with activated CD8^+^ T-cells obtained by allogeneic mixed lymphocyte reaction (MLR) (**Fig. 6C**). To address whether Tregs have potential to induce CTLA4 in CD8^+^ T cells, naïve CD8^+^ T-cells were co-cultured with allogeneic BMDCs in the presence or absence of direct-iTregs *in vitro*. As shown in **new** **Fig. 6D**, direct-iTregs enhanced the CTLA4^+^ expression in CD8^+^ T-cells. These data suggest that CTLA4^+^ tolerogenic CD8^+^ T-cells were developed by the treatment with Tregs in the cardiac grafts.

## Discussion

Tregs are thought to be ideal to prevent allograft rejection during organ transplantation, including heart transplantation, because antigen-specific Tregs are believed to induce antigen-specific tolerance but not systemic immunosuppression. None of the studies has succeeded in generating Tregs specific to a defined peptide ligand that can induce lifelong immunological tolerance. It has been shown that iTregs generated by the direct pathway can prevent the acute phase of cardiac rejection but not chronic phase rejection [32]. Tregs from TCR transgenic mice expanded by auto-antigen-pulsed DCs have been show to reverse hyperglycemia in an animal model of diabetes [33]. This study generated antigen-specific Tregs from TCR transgenic mice; however, this is not applicable to humans. Our method, combining BMDC, TGF-β and an antigen peptide derived from the MHC molecule, is simple and effective for graft-specific Treg generation.

As shown by Joffre et al. [17], we found that direct-iTregs were more efficient than indirect-iTregs for acute phase reaction. The number and purity of Tregs was higher using a direct-Treg injected model, and DC inactivation was also stronger than in indirect-iTreg-injected models at 7 days after transplantation. This was thought to be a result of the difference in infiltration time between indirect-Tregs and direct-Tregs [34].

We found that iTregs induce stronger tolerance than nTregs induced by indirect pathways, although both kept cardiac grafts alive for more than 100 days (**Fig. 3**). This is preferable for clinical application because naïve T cells can be obtained in greater numbers than nTregs. However, this is rather surprising because it has been believed that nTregs are more stable than iTregs. *In vitro*, as well as under lymphopenic conditions, iTregs lost the expression of Foxp3 rapidly and became memory or activated T cells. In contrast to nTregs, adoptively transferred iTregs entirely failed to prevent lethal graft versus host disease (GVHD) [6]. We do not have clear answer yet; however, the expansion rate of the Tregs may be related. We observed that the growth of nTregs was slower than iTregs in the co-culture with the BMDCs, and required higher levels of IL-2. Thus, the survival and/or expansion of iTregs in the graft may be more efficient than those of the nTregs. In addition, TGF-β may exist in high levels in the cardiac graft, which may support iTreg survival. Such environmental factors may contribute to the induction of tolerance by iTregs.

Various mechanisms of prevention of allograft rejection or induction of tolerance by Tregs have been proposed [35]–[39]. *In vivo* control of DC maturation by Tregs is one of the most likely mechanisms. In this theory, Tregs educate DCs to become tolerogenic DCs; then, such tolerogenic DCs generate Tregs from naïve T cells. This positive cycle (so called infectious tolerance [40], [41]) or “immune regulatory feedback loop” can explain the establishment of long-term tolerance, although early exogenous Tregs die at the early stage of transplantation. This idea is supported by the fact that co-transfer of Tregs and tolerogenic DCs induced stronger tolerance to cardiac grafts than individual transfer events [42]. This mechanism is supported by our observation (**Fig 5**) that suppression of DC maturation by Treg therapy. We also observed induction of CD8^+^CTLA4^+^ T cells, which has been shown to be related to tolerance to transplants [43], [44]. A number of reports have already demonstrated that killing activity of CTLs is indeed suppressed by Tregs [45]–[47].

To address the question whether recipient Tregs were really induced by transferred iTregs, we performed preliminary experiments by establishing a model to trance Tregs after transfer. We generated allo-specific Tregs by co-culturing recipient naïve T cells from C57BL6, Ly5.1 mice with BMDCs from donor/recipient F1 BMDC in the presence of TGF-β according to the method reported previously [17]. These Tregs were transferred into Ly5.2 C57BL6 mice transplanted with Balb/c heart. F1 mice were obtained by crossing Balb/c, H-2K^d^ and C57BL6, H-2K^b^. By this method, Tregs were induced by both direct and indirect pathway because F1 BMDCs express both allogenic MHC (H-2K^d^) as well as recipient syngeneic MHC (H-2K^b^). We can distinguish transferred iTregs (Ly5.1) and recipient-derived Tregs (Ly5.2) by flow cytometer. By this method, we have succeeded in counting transferred Tregs and recipient Tregs by FACS on day 7 after cardiac transplantation. In Treg-treated mice, In one cardiac graft, only about 250 Foxp3^+^Ly5.1^+^ Tregs were remained. On the other hand, about 8000 Foxp3^+^Ly5.2^+^ Tregs were present in the graft. In the absence of Treg transfer, less than 1200 Foxp3^+^Ly5.2^+^ Tregs were present in the transplanted heart. Thus, these preliminary data suggests that transferred Tregs cannot survive long in the graft, however, newly generated recipient Tregs are migrated into the graft. Our observations were consistent with the idea of “infectious tolerance”.

Our data suggest that indirect-iTregs induced *in vitro by* a specific antigene are the best Tregs to prevent graft rejection and prolong graft survival. even if antigens of grafts are not known, both direct and indirect pathways can mediate generation of Tregs using F1-DCs [17]. If F1-DCs are not available, cell fusion between donor DCs and recipient DCs may also be applicable. Co-culture of recipient DCs with donor cells may also be applicable, however, the efficiency of presentation of allo-antigens by recipient DCs must be high enough to induce antigen-specific Tregs.

We showed here that iTregs induced *in vitro* can be collected with high purity (>95%) using only surface markers. Moreover, iTregs generated from naïve T-cells were easier to harvest and expand than nTregs. We propose that this model might be useful for clinical Treg cellular therapy in the organ transplantation field.
